# Ginsenoside Rc, as an FXR activator, alleviates acetaminophen-induced hepatotoxicity *via* relieving inflammation and oxidative stress

**DOI:** 10.3389/fphar.2022.1027731

**Published:** 2022-10-07

**Authors:** Yadi Zhong, Yingjian Chen, Zhisen Pan, Kaijia Tang, Guangcheng Zhong, Jingyi Guo, Tianqi Cui, Tianyao Li, Siwei Duan, Xiaoying Yang, Yong Gao, Qi Wang, Dong Zhang

**Affiliations:** ^1^ The Fourth Clinical Medical College of Guangzhou University of Chinese Medicine, Shenzhen, China; ^2^ Science and Technology Innovation Center,Guangzhou University of Chinese Medicine, Guangzhou, China; ^3^ The First Clinical Medical College of Guangzhou University of Chinese Medicine, Guangzhou, China; ^4^ Jiangsu Key Laboratory of Immunity and Metabolism, Department of Pathogen Biology and Immunology, Xuzhou Medical University, Xuzhou, China

**Keywords:** ginsenoside Rc, FXR, acetaminophen, acute liver failure, inflammation, oxidative stress

## Abstract

Acetaminophen (APAP) intake leads to excessive NAPQI deposition, stimulating inflammatory and oxidative stress and causing fatal liver injury. However, the detailed molecular mechanism involved is unknown, and effective therapeutic approaches remain insufficient. In this study, we discovered that treatment with ginsenoside Rc can prevent the inflammatory response caused by APAP and oxidative stress in mouse primary hepatocytes (MPHs), along with the corresponding changes in related genes. Additionally, Ginsenoside Rc effectively alleviates APAP-induced cellular apoptosis and NAPQI accumulation in MPHs. *In vivo*, Ginsenoside Rc administration remarkably attenuates APAP-induced hepatotoxicity, repairing liver damage and improving survival. Moreover, Ginsenoside Rc treatment modulates genes involved in APAP metabolism, leading to a decrease in NAPQI and resulting in the alleviation of fatal oxidative stress and inflammatory response after APAP exposure, along with the expression of their related indicators. Furthermore, our RNA-seq and molecular docking analysis implies that FXR expression and FXR transcriptional activity are stimulated by Ginsenoside Rc treatment. Notably, due to the lack of FXR in mice and MPHs, ginsenoside Rc can no longer play its original protective role against hepatotoxicity and cell damage caused by APAP, and it is difficult to improve the corresponding survival rate and prevent hepatic apoptosis, NAPQI generation, fatal oxidative stress, and the inflammatory response induced by APAP and the expression of related genes. In summary, our results indicate that Ginsenoside Rc could act as an effective FXR activator and effectively regulate FXR-induced antioxidant stress and eliminate inflammation while also having an anti-apoptotic function.

## Introduction

Acetaminophen (APAP) is an antipyretic analgesic that is widely used in clinical practice. It can have a safe therapeutic effect when taken at a reasonable dose (1–4 g/d). However, if the dosage of APAP is too high, it can easily to cause acute liver injury (Tsai et al., 2015) or even an emergency outbreak of FLF (Fatty Liver Foundation) liver failure (Soeda et al., 2014). In China, APAP is one of the three main causes of acute liver failure (Gao et al., 2020b). APAP is metabolized by hepatocyte cytochrome P450 (CYP450), and converted to the active intermediate N-acetyl-p-benzoquinimide (NAPQI). Too much APAP causes NAPQI to consume all of the glutathione (GSH). It combines to form APAP adducts, causing mitochondria to lose their normal functions, produce reactive oxygen species (ROS) and release mitochondrial cell death factors, leading to liver necrosis (Chen D. et al., 2019; Chen et al., 2020). As the only approved antidote for AILI (Acetaminophen-induced liver injury), N-acetyl-cysteine (NAC) can cause nausea and other adverse reactions, and its utility is limited to the early days of treatment. Later treatment with NAC may even damage liver regeneration (Yin et al., 2010; Gao et al., 2020a). Therefore, it is necessary to find a new and effective treatment method for acute liver injury caused by APAP. Reducing APAP-induced oxidative stress and inflammation may be an effective strategy.

The nuclear receptor farnesoid X receptor (FXR; NR1H4) potently regulates lipid, bile acid (BA), and glucose metabolism. Bile acids regulate the bile acid balance, inflammation, and lipid and glucose metabolism by activating the nuclear receptor FXR and membrane G protein-coupled receptor TGR5. Cholestasis can lead to the accumulation of BAs in the liver. FXR is not only expressed in the liver, but also exists in the intestine to control the reabsorption of bile acids, acting as a key regulator of bile acid enterohepatic circulation (Anakk et al., 2011; Baghdasaryan et al., 2011). It also plays an essential role in the metabolism of xenobiotics (Fiorucci et al., 2012). FXR induces transcriptional repression with small heterodimer partners (SHP), thereby inhibiting the transactivation of human NTCP and CYP7A1 genes. This mechanism can prevent bile acid overload and liver cell damage during cholestasis (Bechmann et al., 2013). In addition, FXR combines with bound bile acids to induce fibroblast growth factor (FGF)-15 to reduce the transcription of cytochrome P450 enzyme (Cyp7a1) in hepatocytes, further inhibiting the *de novo* synthesis of bile acids (Hartmann et al., 2018). Previous studies have shown that FXR and SHP strictly regulate the physiological concentration and homeostasis of bile acids through a multistep feedback loop, and ultimately inhibit cytochrome P450 (CYP7A1) (Anakk et al., 2011). Therefore, FXR may be a target protein mediating drug-induced liver injury and play an anti-inflammatory role to help inhibit the development of drug-induced liver injury.

Ginseng, a precious medicinal material that has been widely used in Asian countries for thousands of years, can produce a variety of medicinaleffects in the field of disease treatment, including antitumor, antidiabetic, antifatigue, antioxidation, heart protection, and immune stimulation and neuroprotective effects. Ginsenoside is the main active ingredient responsible for its pharmacological effects (Kim et al., 2013; Lee et al., 2019). Ginseng can also be used as a chemopreventive agent an adjuvant therapy (Jeon et al., 2020). Previous research results have shown that ginsenoside Rc targets mitochondrial functions to increase the ATP content, remove free radicals and return to normal oxygen consumption levels. In addition, studies have found that ginsenoside Rc can stimulate and accelerate energy metabolism and fight oxidative damage, inflammation, cancer, aging, and metabolic diseases by activating SIRT1 (Huang et al., 2021). This study can treat APAP-induced liver injury by intervening inflammation, oxidative stress, and liver regeneration, which has the characteristics of multi-pathway and multi-target, and can play a better role in liver protection and liver protection. At the same time, it was found that ginsenoside Rc had a preventive protective effect on APAP-induced liver injury, and its mechanism was related to anti-oxidative stress, anti-apoptosis of hepatocytes, inhibition of the expression of inflammation-related factors, and regulation of APAP metabolism and transport *in vivo*. And we will further study the protective effect of FXR nuclear receptor, a key target of liver metabolism regulation, on APAP-induced acute liver injury, acute hepatocyte injury, oxidative stress, and inflammatory response, identify key genes regulating antioxidant genes and explore new potential drug targets, and improve the related mechanism of FXR on liver protection. To provide ideas and basis for the efficacy study and drug development and utilization of ginsenoside Rc.

## Materials and methods

### Reagents

Ginsenoside Rc (CAS.No11021-14-0) (purity 98.8%) and acetaminophen (APAP) were purchased from Shanghai Yuanye Biotechnology Co., Ltd. (Shanghai, China) and Aladdin Chemical Company (Shanghai, China).

### Animal experiment

Male C57BL/6 mice weighing 20–25 g and aged 6 weeks were purchased from Model Animal Research Center of Guangzhou University of Traditional Chinese Medicine (CertificateSCXK 2018-0034; Guangzhou, China) (Chen L. et al., 2019). FXR knockout (FXR^−/−^) mice were kindly sponsored by Changhui Liu and were originally purchased from Jackson Laboratory (Bar Harbor, ME, United States) (Liu et al., 2020). All the mice were housed in individually ventilated cages and were kept on a 12-h light dark cycle at all times. All the animal experiments carried out were submitted to and approved by the Animal Ethics Committee of Guangzhou University of Traditional Chinese Medicine. WT and FXR^−/−^ mice received daily injections of ginsenoside Rc intraperitoneally at a dose of 5 mg/kg or 10 mg/kg. The control group was given normal saline (Control). The injection cycle was 10 days. On the 11th day, all observation groups were fasted overnight, and then injected with APAP dissolved in 60°C normal saline, 300 mg/kg. The injection site was the abdominal cavity, and the control group did not undergo this operation. All mice were sacrificed 10 h after APAP injection. Fresh liver tissue from the mice was then extracted for further studies. The total glutathione/-oxidized glutathione assay kit (Jianchen Bioengineing China) was used as the measurement tool for serum ALT and AST concentrations and GSH. TNF-α (Ruixinbio, Cat.#RX-D202412M), IL-6 (Ruixinbio, Cat.#RX-D203063M) and IL-1β (Ruixinbio, Cat.#RX-D203049M), NAPQI (Cat.#SU-B28212) were measured using an ELISA (Ruixinbio, Quanzhou, China). The One step TUNEL Apoptosis Assay Kit (MA0224-1) (meilunbio, Shanghai, China) was also used.

### Mouse survival experiment

In experiments on drug-induced liver injury, 300 mg/kg of APAP was injected to mice prior to the injection of different doses of Rc. In experiments conducted to test the survival rate, the time to death of mice within 72 h was recorded.

### Cell protocols

Mouse primary hepatocytes (MPHs) were isolated and cultured in RPMI-1640 medium with 10% fetal bovine serum, 0.1 mg/ml streptomycin, and 100 units/ml penicillin as described above. (Liang et al., 2021b; Yang et al., 2020). After 24 h of incubation, the hepatocytes were treated with 10 mM APAP for an additional 24 h.

### CCK-8 assay

A 96-well plate was used as a culture tool for MPHs, and the concentration of 5 × 104/ml was repeated every five wells. After 6 h of cell incubation, a new culture base was formed, that is, configure various concentrations of ginsenoside Rc were configured, and the culture time was 24 h. The medium was removed and the culture was transferred to 100 μl of new medium containing CCK-8 (1640 medium and CCK-8 solution in a ratio of 10:1) for incubation, and then the OD value was measured at 450 nm (Liang et al., 2021b; Yang et al., 2020).

### Measurement of ROS

Briefly, after incubating MPH and Rc together, they were co-incubated with APAP for 24 h. In the next step, they were placed in DCFH-DA at concentration of 10 μm and incubated again at 37°C for half an hour while avoiding light. Finally, the cells were washed three times with PBS, and the fluorescence emissions of the cells were observed by a fluorescence microscope. Then, the reactive oxygen species in the body were analyzed (Liang et al., 2021b; Yang et al., 2020). DCFH-DA (10 mM) was injected into mice through the tail vein, and again 1 h later. After death, liver tissue was extracted. The luciferase reporter gene for ROS levels in the extracted liver was detected by Berthold technology LB983 NC100.

### TUNEL assay

Liver tissue was fixed with 4% paraformaldehyde solution, further sectioned at 4 μm, and embedded in paraffin. TUNEL levels were determined with the TUNEL Apoptosis Assay Kit (MA0224-1, meilunbio) according to the manufacturer’s instructions. Imaging was achieved with the aid of a fluorescence microscope (Leica Microsystems Ltd., Wetzlar, Germany).

### Histology and immunofluorescence

Liver tissue was fixed with 10% paraformaldehyde, sectioned at 5 μm, and embedded in paraffin. After routine hematoxylin and eosin (H&E) staining, the sections were placed under a slide scanner (Panoramic MIDI 1000×) at the Lingnan Medical Research Center of Guangzhou University of Traditional Chinese Medicine for further observation. Immunofluorescence was performed according to current protocols. Liver sections or MPH were incubated with NFkB-p65 (Affinity, United States) or primary antibodies F4/80, CD11b at 4°C overnight. After being washed three times with PBS, sections were incubated with a secondary antibody (Abclonal, Wuhan, China) at room temperature for 40 min. After being washed 5 times with PBS, images were observed using a fluorescence microscope (Nikon 1000×) from the Science and Technology Innovation Center of Guangzhou University of Chinese Medicine (Liu et al., 2020; Sun et al., 2021).

### Western blotting

MPHs (WT and KO) that had been infected with ginsenoside Rc were exposed to APAP (10 mM) for 24 h. Proteins were extracted from Ginsenoside Rc-treated MPH cells, and the concentrations were determined using a BCA assay kit produced by Biyuntian Technology, Beijing, China. Equal amounts of protein (60 μg) were separated with 10% SDS-polyacrylamide gels, and the fractions were transferred to PVDF membranes. The antibodies used were specific for rabbit anti-Bax (#T-40051, abmart) and rabbit anti-Bcl2 (#T-40056, abmart), rabbit anti-FXR (25055-1-AP, Proteintech), and rabbit anti-β-actin (AC026, abclonal).

### Quantitative PCR

Total mRNA from MPH or liver tissue was extracted using TRIzol reagent. Reverse transcription was accomplished using a high-capacity cDNA reverse transcription kit produced by Applied Biomaterials Canada. qPCR analysis of cDNA was performed with the help of PowerUpTM SYBRTM Green Master Mix from Aibotech Biotech, Wuhan, China. The normalization of all genes was completed using *β*-actin, and the specific primer sequences are shown in Supplementary Table S1.

### Statistical analysis

The results for all the figures and text are presented as the mean ± SEM. Data were evaluated by using Student’s t-test and one-way analysis of variance (ANOVA) to identify statistical differences between groups. This was followed by an analysis with GraphPad Prism (version 8.0) to determine statistical differences between groups. The post-hoc Tukey’s test was performed on the results after multiple group comparisons. Survival was repeatedly measured by means of a two-way ANOVA and plotted accordingly. *p*-values < 0.05 were deemed significant.

## Results

### Ginsenoside Rc attenuates APAP overdose-induced ALI by restraining oxidative stress, and inflammation apoptosis in MPHs

**FIGURE 1:**
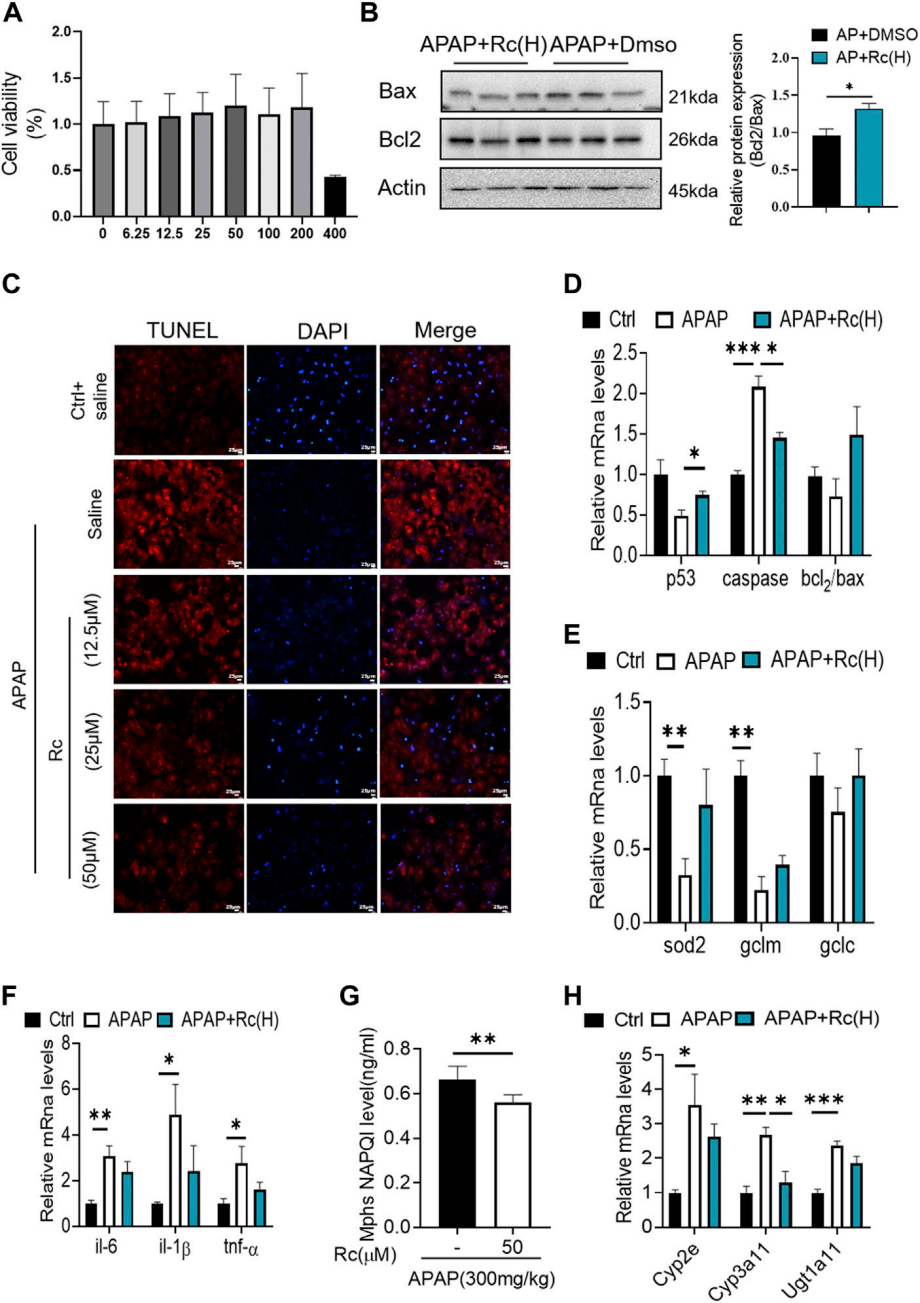
Ginsenoside Rc suppressed APAP-induced hepatocellular damage by reducing oxidative stress, and inflammation in MPHs. **(A)** CCK8 assay; **(B)** Ginsenoside Rc treatment inhibits the expression of Bcl2 protein and restrains the expression of bax in MPHs. **(C)** The TUNEL assay was performed to measure the apoptosis effect in MPHs. **(D)** Relative expression of mRNA associated with antiapoptosis (P53, Caspase, Bcl2/Bax);**(E)** Relative expression of mRNA associated with oxidative stress (Sod2,Gclc, Gclm); **(F)** Relative expressions of mRNA associated with inflammation (Il-6, Il-1β, TNF-α); **(G)** The ELISA assay showed that Ginsenoside Rc treatment decreased NAPQI levels in MPHs; **(H)** Relative expression of RNA associated with APAP-metabolizing enzymes (Cyp3a11,Cyp2E,Ugt1a11); Data are means ± SEM; n = 3-6/group.*, *p* < 0.05, **, *p* < 0.01, ***, *p* < 0.001.

To investigate whether Ginsenoside Rc can attenuate APAP overdose-induced ALI in MPHs, we established the APAP (10 mM)-incubated MPH model. As shown in Figure 1A, Ginsenoside Rc dose-dependently alleviated APAP-induced hepatocyte damage, with a maximal effect at 50 µM (h). Next, we tested Bcl2 and Bax expression in MPHs using Western immunoblotting. After APAP-induced injury in MPHs, as can be seen in (Figure 1B), the expression of the antiapoptotic gene Bcl2 was inhibited to a certain extent, and the expression of the related apoptosis gene Bax was inhibited to a greater extent, thereby significantly reducing the relative mRNA transcription level of Bcl2/Bax. However, Ginsenoside Rc treatment can reverse the downregulation of the mRNA relative expression level of Bcl2/Bax (Figures 1C,D). Moreover, TUNEL staining after high-dose Ginsenoside Rc treatment compared the number of red fluorescent label-positive apoptotic cells was the injury model group, showing a significant decrease.

In order to further analyze the oxidative stress effect of ginsenoside Rc on APAP-induced liver injury, we further explored the expression of Sod2, Gclc and Gclm at the mRNA level. These are key factors of antioxidant defense, APAP treatment decreased the mRNA transcription level of Sod2 (Figure 1E). This indicated that Ginsenoside Rc could significantly improve the antioxidant defense system function of the damaged liver, alleviating oxidative stress injury. In addition, Ginsenoside Rc administration significantly reduced NF-KB levels in APAP-induced MPHs (Supplementay Figure. S1). Additionally, genes involved in inflammation such as IL-6, TNF-α, and Il-1β were significantly decreased after Ginsenoside Rc treatment (Figure 1F).

Since excess APAP needs to be metabolized by cytochrome P450 (CYP) CYP2E1 and CYP3A4 to the highly reactive metabolite N-acetyl-p-benzoquinoneimide (NAPQI), we further evaluated the expression levels of NAPQI and CYP in the liver, including CYP2E1, CYP3A11, and Ugt1a11 in MPHs. We found that, after Ginsenoside Rc treatment, the level of NAPQI decreased (Figure 1G), and the gene expression levels of these enzymes were significantly lower in the MPHs with ALI than in the MPHs without APAP treatment (Figure 1H). Interestingly, treatment with Ginsenoside Rc further significantly decreased the expression levels of these genes. In conclusion, it can be seen from these data that ginsenoside Rc can reduce ALI caused by excessive APAP by inhibiting oxidative stress, and inflammation apoptosis in MPHs.

### Ginsenoside Rc alleviated hepatic damage in ALI mice by regulating oxidative stress and drug metabolism

**FIGURE 2:**
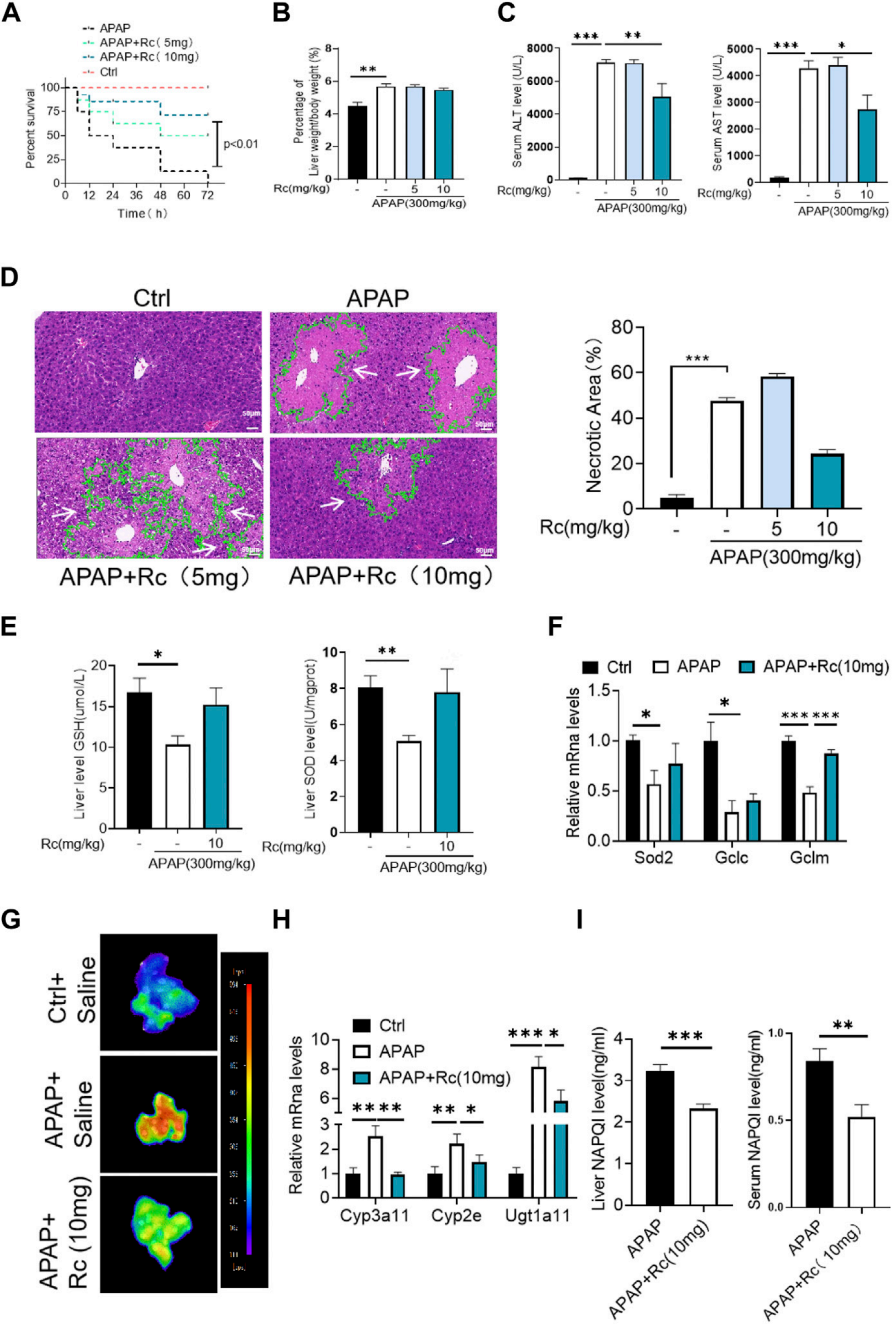
Ginsenoside Rc alleviated hepatic damage in ALI mice by regulating oxidative stress. **(A)** The survival rate of mice (with/without Ginsenoside Rc treatment) after intraperitoneal injection of APAP for 72 h**(B)** The ratio of liver weight/body weight; **(C)** Serum ALT and AST levels; **(D)** H&E staining of livers (1000×); **(E)** Hepatic GSH and SOD levels were reduced in APAP-induced mice after Ginsenoside Rc treatment; **(F)** Expression of mRNA levels of oxidative stress genes (Sod2, Gclc, Gclm); **(G)** Hepatic ROS levels were decreased in APAP-induced mice after Ginsenoside Rc treatment; **(H)** Relative expression of mRNA associated with APAP-metabolizing enzymes (Cyp3a11, Cyp2E, Ugt1a11); **(I)** The ELISA assay showed Ginsenoside Rc treatment decreased NAPQI levels in APAP-administrated mice. Data are means ± SEM; n = 5-8/group. *,*p* < 0.05, **; *p* < 0.01, ***; *p* < 0.001.

Since ginsenoside Rc can significantly contribute to oxidative stress in MPH, it was hypothesized that ginsenoside Rc may be ameliorated in ALI mice. Therefore, mice were injected with different doses of ginsenoside Rc daily for 10 days. As shown in Figures 2A–C, after the mice received ginsenoside Rc treatment, and then received APAP treatment, they were fully protected, and at the same time, the serum ALT and AST levels were significantly reduced, and lethality was significantly lower. The improved survival rate could be validated by the APAP dose (300 mg/kg). In addition, after receiving ginsenoside Rc treatment, the rate and extent of necrosis of the centrilobular liver induced by APAP excess were significantly reduced, as shown by the hematoxylin and eosin (H&E) staining results in Figure 2D. Additionally, from the graph above, we can see that Ginsenoside Rc treatment enhanced GSH (Figure 2E) and increased the antioxidant gene expression, while the hepatic NAPQI levels were notably reduced (Figure 2I). Furthermore, Ginsenoside Rc significantly mitigatied the otherwise APAP-induced hepatic ROS overproduction (Figure 2G) and induced changes in the expression of genes involved in oxidative stress (Figure 2F), which suggests that Ginsenoside Rc can attenuate APAP excess-induced ALI by enhancing antioxidant defenses.

### Treatment with ginsenoside Rc effectively alleviates APAP overdose-induced inflammation and apoptosis

**FIGURE 3:**
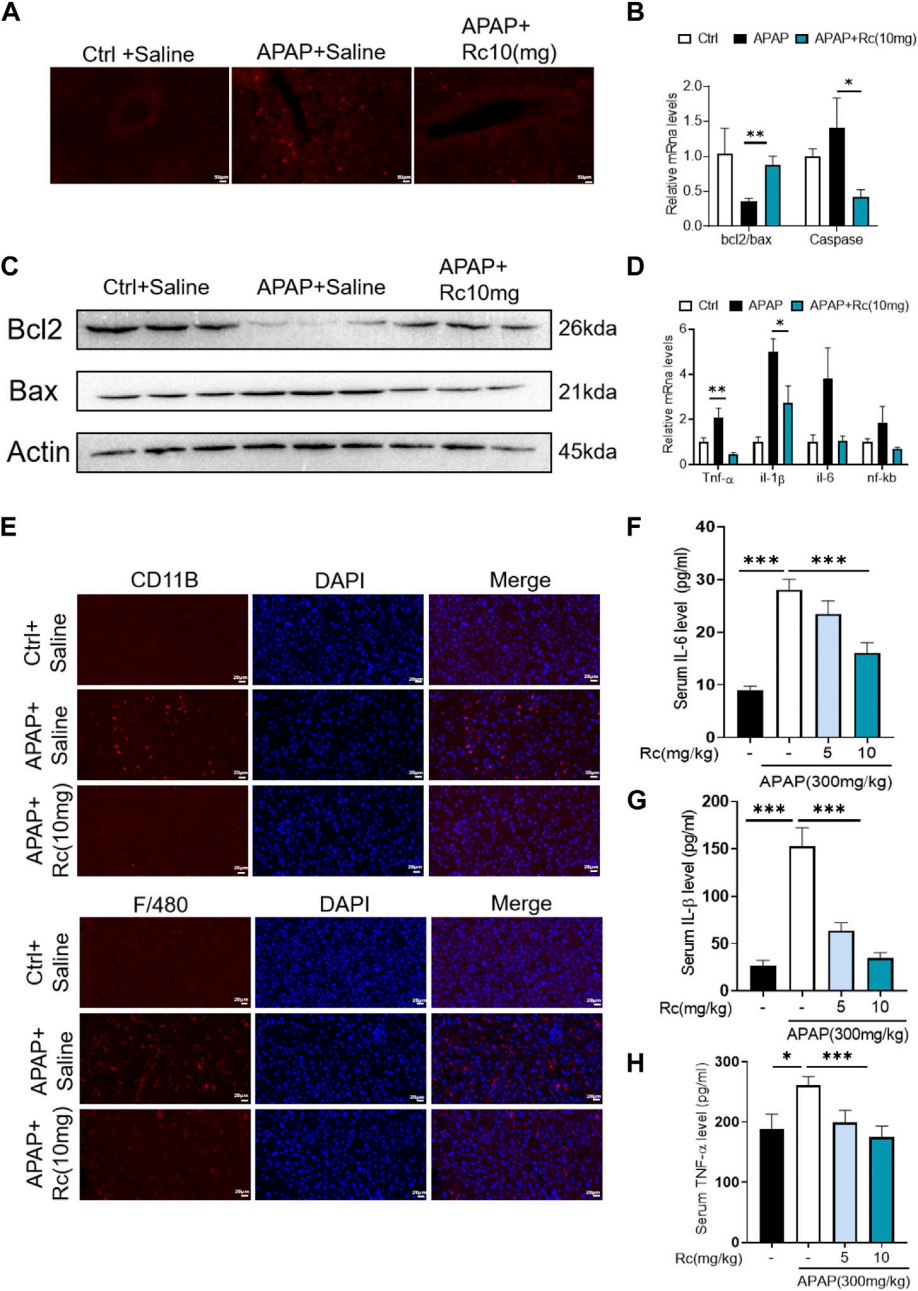
Treatment with Ginsenoside Rc Effectively Alleviates APAP Overdose-Induced Inflammation and antiapoptosis **(A)** The TUNEL assay was performed to measure the apoptosis effect in APAP-induced mice; **(B)** Relative expression of mRNA associated with antiapoptosis (Caspase, Bcl2/Bax); **(C)** Ginsenoside Rc treatment inhibits the expression of the Bcl_2_ protein and restrains the expression of Bax in mice; **(D)** Relative expression of mRNA associated with inflammation (Il-6, Il-1β, TNF-α); **(E)** The protein levels of F4/80 and CD11b were determined using immunofluorescence (1000X). The nuclei were stained with DAPI; **(F–H)** Serum pro-inflammatory cytokines, including TNF-α, IL-1β, and IL-6, were decreased in APAP-induced mice after Ginsenoside Rc treatment. Data are means ± SEM; *n* = 5-8/group. *, *p* < 0.05, **; *p* < 0.01, ***; *p* < 0.001.

APAP overdose generates adducts with proteins in hepatocytes, which can lead to cell apoptosis. In the control injury model group, high-dose ginsenoside Rc treatment could significantly reduced the leverl of positive cell apoptosis marked by red fluorescence in TUNEL staining (Figure 3A). As shown in Figures 3B,C, APAP upregulates Bax and significantly reduces Bcl-2 expression at the mRNA level and in protein in liver tissue; however, ginsenoside Rc treatment can significantly protect mice from apoptosis induced by APAP excess, as TUNEL levels in liver were significantly reduced.

Acute liver injury can cause severe oxidative stress and trigger the liver’s innate immune system, promoting the recognition and presentation of antigens by various immune and non-immune cells in liver tissue, thereby inducing persistent inflammatory responses and hepatocyte damage. We next analyzed serum levels of the proinflammatory cytokines TNF-α, IL-1β, and IL-6 and hepatic mRNA levels of TNF-α, IL-1β, and IL-6 in APAP-induced liver injury mice. We found that the markers of inflammatory monocytes F4/80 and CD11b showed significant increases. As shown in Figure 3E, mice with liver injury due to APAP showed higher levels of the serum, liver inflammatory factors TNF-α, IL-1β, and IL-6 at the mRNA transcription level compared with the control group (Figures 3D,F–H). However, ginsenoside Rc treatment effectively suppressed the elevation of these inflammatory factors in the serum, and treatment with ginsenoside Rc significantly reduced hepatic transcriptation levels of TNF-α, IL-1β, and IL-6. It can be seen that ginsenoside Rc can effectively inhibit the inflammatory response induced by APAP-induced acute liver injury in mice.

### FXR serves as a candidate target for ginsenoside Rc against ALI

**FIGURE 4:**
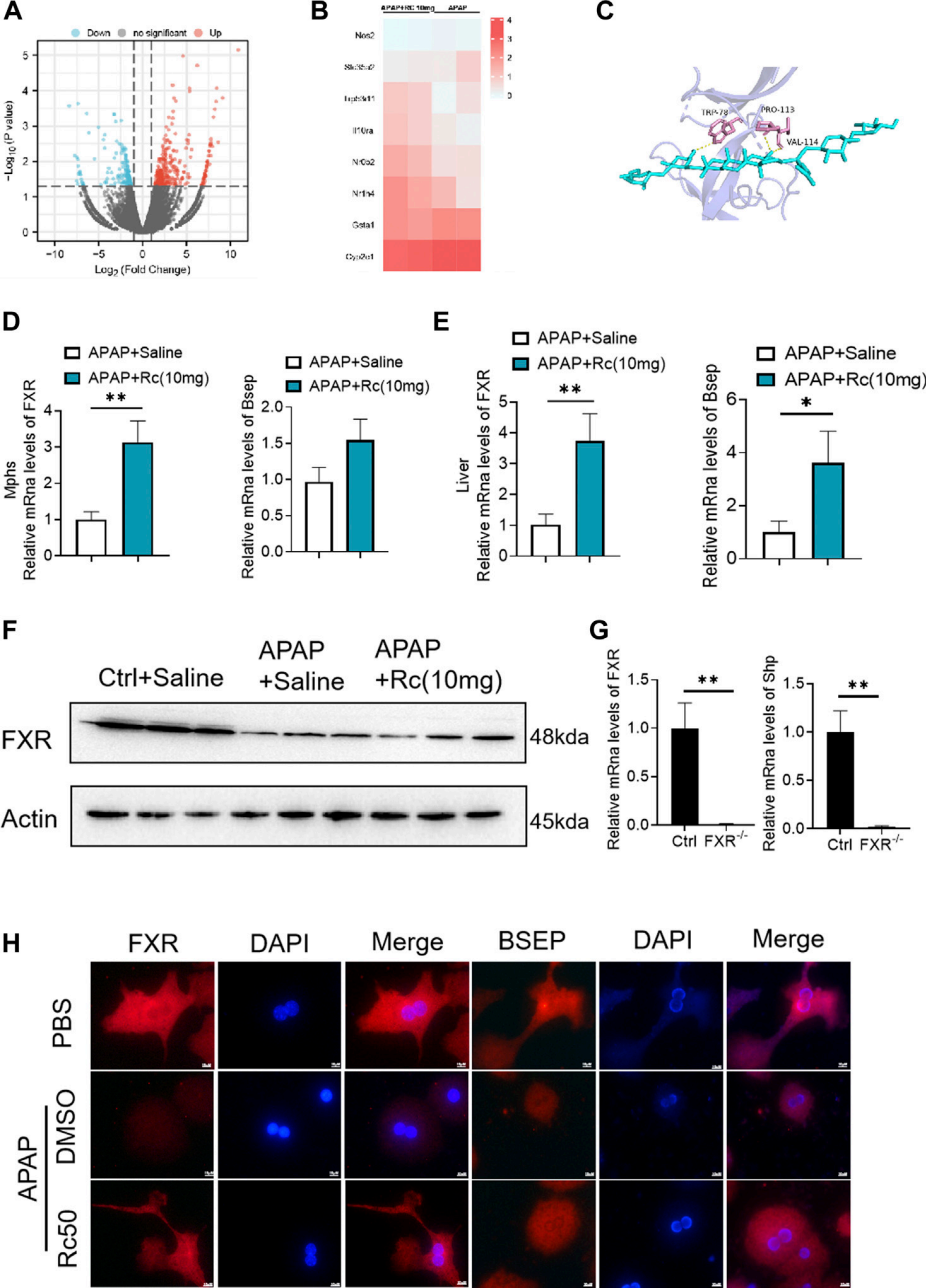
FXR Serves as a Candidate Target for Ginsenoside Rc against ALI. **(A,B)** Heatmap of RNAseq on the liver from Ginsenoside Rc (10 mg/kg) - or saline-treated APAP-induced mice showing the changes in FXR and genes associated with APAP-metabolizing enzymes and antioxidant activity. **(C)** Ginsenoside Rc and FXR are bonded by hydrogen bonds; **(D,E)** Treatment of Ginsenoside Rc reduced the impairment in mRNA expression of FXR and FXR-dependent downstream target genes in liver tissue and MPHs. The mRNA levels of FXR were determined by qRT-PCR. **(F)** Treatment of Ginsenoside Rc reversed the downregulation of the protein levels of FXR in the liver tissue induced by APAP. **(G)** Relative expression of FXR mRNA in FXR^−/−^ and FXR^+/+^ mice. **(H)** The immunofluorescence data indicate that the protein levels of FXR and BSEP were increased in APAP-induced mice after Ginsenoside Rc treatment. (magnification: 1000x). The nucleus was stained with DAPI. Data are means ± SEM; n = 5-8/group. *,*p* < 0.05, **; *p* < 0.01, ***; *p* < 0.001.

In order to further analyze the antagonism mechanism of ginsenoside Rc against ALI, we carried out a transcriptional analysis of liver tissue. Consistent with the above research conclusions, mice treated with APAP showed significant inhibition of the expression of FXR and its target genes SHP (NROB2) and BSEP (ABCB11). Genes involved in the antioxidation pathway, such as INOS(nos2) and genes related to APAP-metabolizing enzymes, such as UGT1(Slc35a2) and the CYP2E1 gene had decreased concentrations after exposure to Ginsenoside Rc, which has been shown to act by modulating hepatic metabolic enzymes and oxidative stress in ALI induced by APAP (Figures 4A,B). In addition, we carried out a more in-depth docking analysis of ginsenoside Rc and FXR, and the data show that the hydrophobic interaction can lead to a greater binding affinity (Figure 4C). In conclusion, this study concludes that FXR is likely to be a new candidate target for altered expression.

After qPCR and the immunofluorescence analysis, it was again confirmed that Ginsenoside Rc can significantly increase the expression of FXR *in vivo* and *in vitro* (Figures 4D,E,H). As expected, Ginsenoside Rc increased FXR *via* inhibition of the expression levels of SHP (Supplementary Figure S2) and BESP. APAP significantly reduced FXR expression at the protein and mRNA levels in liver tissue (Figures 4F, Supplementary Figure S6). However, mice treated with ginsenoside Rc can effectively reduce the damage caused by excessive APAP. We verified the knockout of FXR using qPCR and genotyping (Figures 4G, Supplementary Figure S3).

### The absence of FXR abrogated the hepatoprotective effects of ginsenoside Rc against APAP overdose-induced ALI in MPHs

**FIGURE 5:**
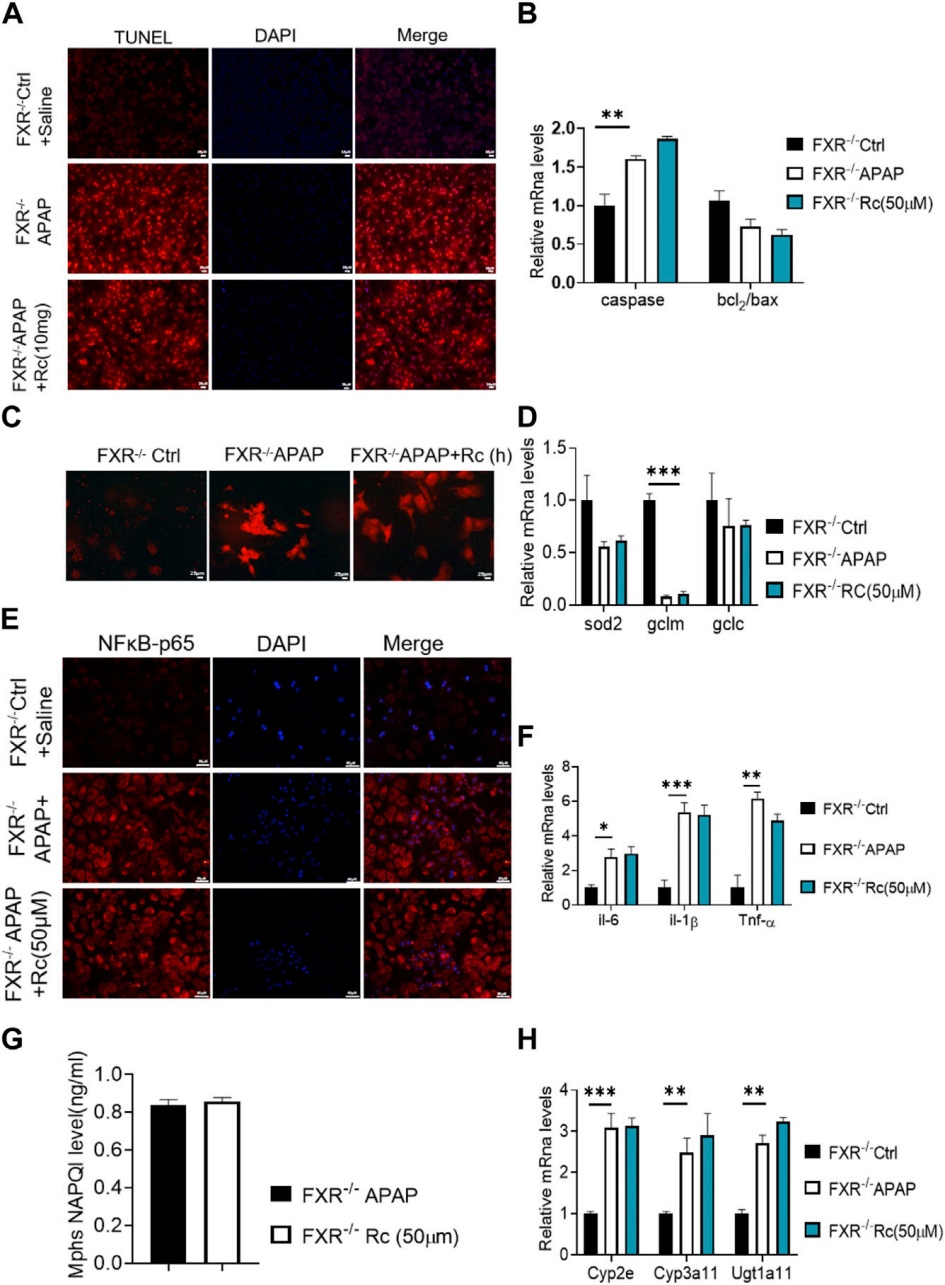
The absence of FXR abrogated the hepatoprotective effects of Ginsenoside Rc against APAP overdose-induced ALI in MPHs. **(A)** The TUNEL assay was performed to measure the apoptosis effect in FXR^−/−^ MPHs;**(B)** Ginsenoside Rc administration did not alter the genes involved in apoptosis in FXR^−/−^ MPHs; **(C)** ROS fluorescence in FXR^−/−^ MPHs; **(D)** Expression of mRNA levels of oxidative stress genes (Sod2,Gclc, Gclm) in FXR^−/−^ MPHs; **(E)** Immunofluorescence for NFκB- p65 (400×); **(F)** Ginsenoside Rc administration does not alter the genes involved in inflammation in FXR^−/−^ MPHs (Il-6, Il-1β, Tnf-α); **(G)** The ELISA assay showed that Ginsenoside Rc treatment does not alter the NAPQI levels in FXR^−/−^ MPHs. **(H)** Ginsenoside Rc administration does not alter the genes involved in APAP-metabolizing enzymes in FXR^−/−^ MPHs (Cyp3a11, Cyp2E, Ugt1a11); Data are means ± SEM; n = 5-8/group. *,*p* < 0.05, **; *p* < 0.01, ***; *p* < 0.001.

As can be seen from Figure 5A, the TUNEL levels in FXR^−/−^ MPH were not significantly changed after ginsenoside Rc treatment with or without APAP overdose. Furthermore, ginsenoside Rc was unable to remodel the expression of Bcl-2 and Bax in FXR^−/−^ MPHs (Figure 5B). However, in the absence of the FXR gene, the ROS-suppressive effect of ginsenoside Rc production was reduced with APAP exposure (Figure 5C), and even ginsenoside Rc treatment did not alter the antioxidant-determining effect of the FXR^−/−^ MPHs ability genes (Figure 5D). In FXR^−/−^ MPHs, although ginsenoside Rc also inhibited liver inflammation induced by APAP excess (Figure 5E), the effect was significantly weakened. APAP excess does not significantly alter the FXR^−/−^ MPH of TNF-α, IL-1β, and IL-6 (Figure 5F). The level of NAPQI and its related genes also indicated that there are no differences in APAP-metabolizing enzymes (Figures 5G,H).

In summary, treatment with ginsenoside Rc did not alter liver genes involved in beta oxidation or APAP-metabolizing enzymes in FXR^−/−^ MPHs. Taken together, these data suggest that, in APAP-induced MPH injury, FXR is clearly dependent on ginsenoside Rc.

### Ginsenoside Rc failed to alter the APAP-induced ROS burden in FXR-deficient mice

**FIGURE 6:**
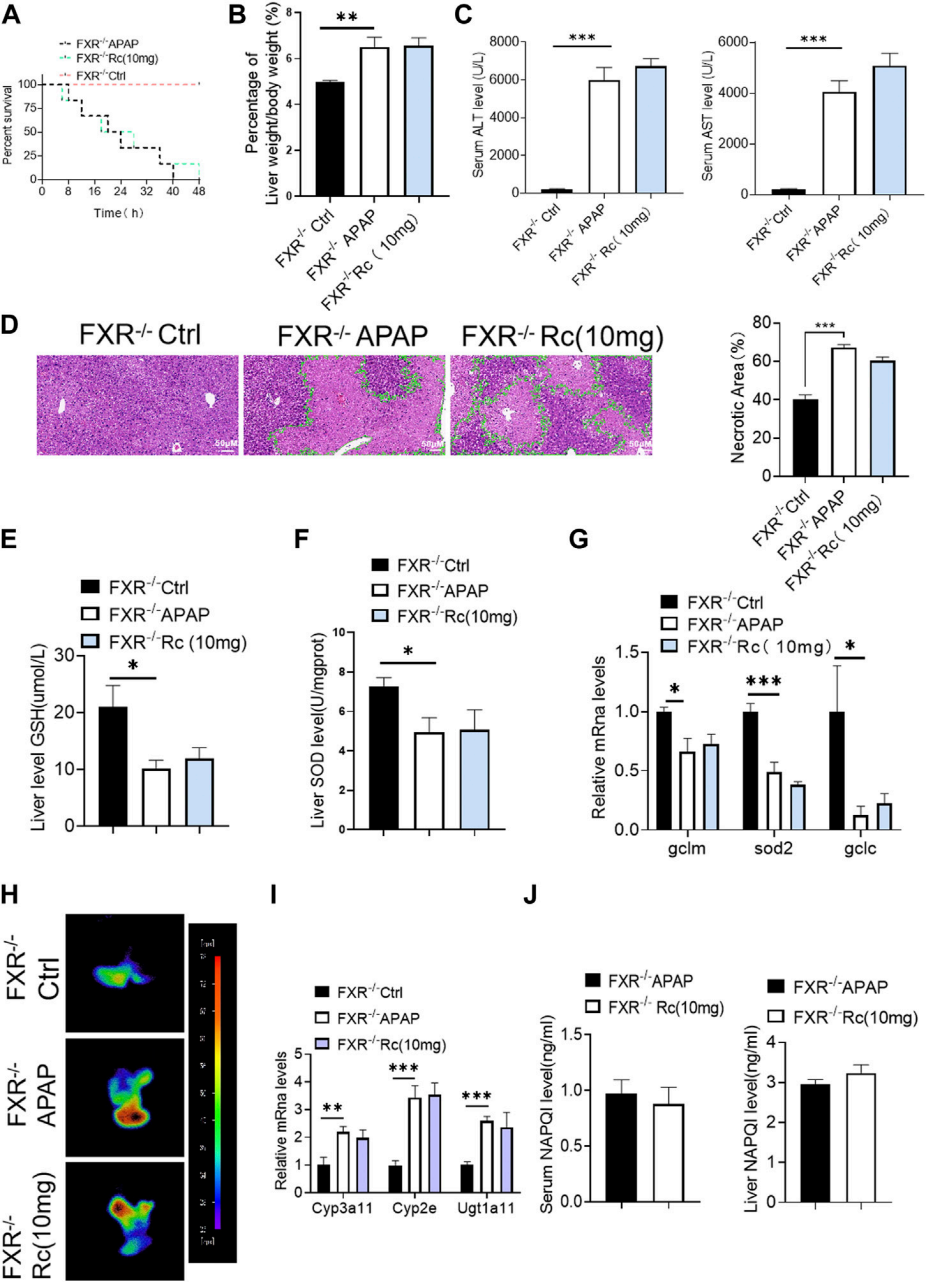
Ginsenoside Rc failed to alter the APAP-induced ROS burden in FXR^−/−^ mice. **(A)** The survival rate of FXR^−/−^ mice (with/without Ginsenoside Rc treatment) after intraperitoneal injection of APAP for 72 h. **(B)** The ratio of liver weight/body weight in FXR^−/−^ mice; **(C)** Serum ALT and AST levels in FXR^−/−^ mice; **(D)** H&E staining of FXR^−/−^ livers (1000×); **(E–G)** Ginsenoside Rc administration did not alter the GSH or SOD levels in FXR^−/−^ mice or the oxidative stress genes (Sod2, Gclc, Gclm); **(H)** ROS fluorescence in FXR^−/−^ mice; **(I)** Ginsenoside Rc administration did not alter the genes associated with APAP-metabolizing enzymes in FXR^−/−^ mice (Cyp3a11, Cyp2E, Ugt1a11); **(J)** The ELISA assay showed that Ginsenoside Rc treatment does not alter the NAPQI levels in FXR^−/−^ mice. Data are means ± SEM; n = 5-8/group. *,*p* < 0.05, **; *p* < 0.01, ***; *p* < 0.001.

Similar to the elimination of APAP-induced production in KO MPHs by ginsenoside Rc, there was no significant difference in the survival rate of FXR^−/−^mice (Figure 6A), and Ginsenoside Rc treatment failed to alter the ratio of liver tissue to body weight in FXR-deficient mice (Figure 6B). Additionally, in the absence of hepatic FXR, ginsenoside Rc treatment failed to alter hepatic AST and ALT levels (Figure 6C). Moreover, it could not effectively improve the necrotic area of hepatic histopathology in FXR^−/−^ mice (Figure 6D).

Moreover, hepatic antioxidants, such as SOD and GSH, were not altered by ginsenoside Rc in the absence of FXR (Figures 6E,F), as was shown for the expression levels of these antioxidants at the mRNA level (Figure 6G). Ginsenoside Rc loses a significant protective effect against APAP-induced damage due to intrahepatic ROS production in mice with FXR (Figure 6H). Likewise, the NAPQI level and the genes related to APAP-metabolizing enzymes were not shown to be altered in FXR^−/−^ mice, even after Ginsenoside Rc treatment (Figures 6I,J).

### Hepatic FXR deficiency diminished the effects of ginsenoside Rc against APAP-induced inflammation and antiapoptosis

**FIGURE 7:**
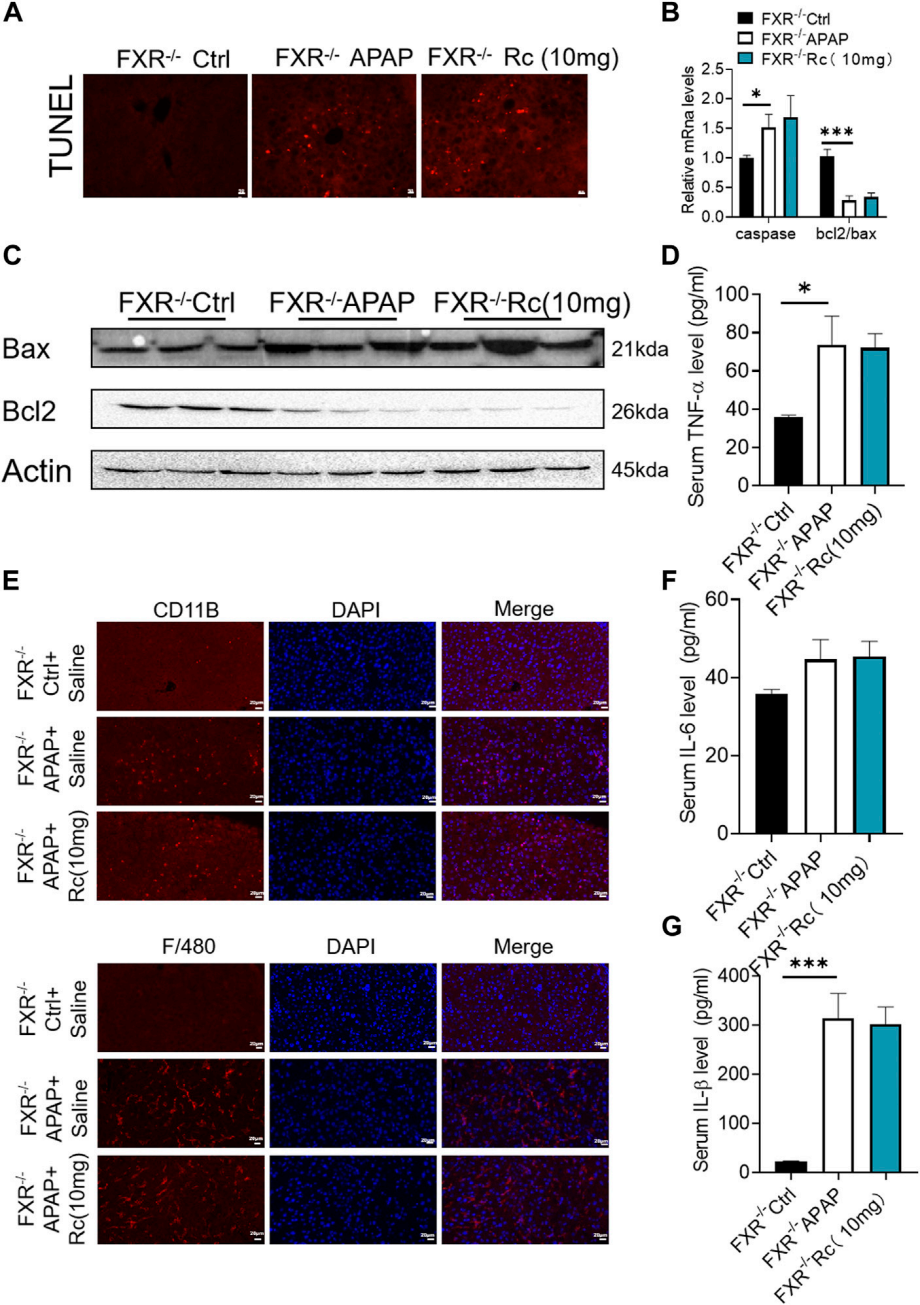
FXR^−/−^ diminished the effects of Ginsenoside Rc against APAP-induced by inflammation and antiapoptosis. **(A)** The TUNEL assay was performed to measure the apoptosis effect in FXR^−/−^ mice; **(B,C)** Ginsenoside Rc administration did not alter the genes involved in apoptosis in FXR^−/−^ mice; **(D,F,G)** Serum pro-inflammatory cytokines, including Tnf-α, IL-1β, and IL-6, were not altered in APAP-induced FXR^−/−^ mice after Ginsenoside Rc treatment; **(E)** The protein levels of F4/80 and CD11b were determined using immunofluorescence (1000X). The nuclei were stained with DAPI; Data are means ± SEM; *n* = 5-8/group. *,*p* < 0.05, **; *p* < 0.01, ***; *p* < 0.001.

In the earlier experiments, Ginsenoside Rc could not improve apoptosis and inflammation dependent on FXR under conditions of APAP-induced cellular damage in FXR^−/−^MPHs. We further detected its effect *in vivo*. It was observed that ginsenoside Rc treatment with or without APAP overdose did not significantly alter TUNEL levels in FXR^−/−^ mice (Figure 7A). Moreover, Ginsenoside Rc was unable to alter the expression of Bcl-2 and Bax at the mRNA and protein levels (Figures 7B,C). The immunofluorescence experiments of F4/80 and CD11b showed that the improvement of hepatic inflammation with Ginsenoside Rc was abolished in FXR^−/−^mice (Figure 7E). Moreover, for FXR^−/−^ mice, it was also difficult for ginsenoside Rc treatment to produce a significant inhibitory effect on liver inflammation caused by APAP excess. TNF-α, IL-1β and IL-6 levels in FXR^−/−^ mice were not specifically changed after overdose of APAP (Supplementary Figure S4). The above-mentioned gene expression and Bax protein concentration were not significantly altered in FXR^−/−^ mice after ginsenoside Rc treatment (Figures 7D,F–G).

It was shown that Ginsenoside Rc lost its significant protective effect against liver damage in FXR^−/−^ mice compared with WT mice, suggesting that FXR plays an important regulatory role in the protection of ginsenoside Rc and APAP-induced acute liver injury.

## Discussion

Acetaminophen (APAP), known as paracetamol, is commonly used as an analgesic, anti-inflammatory, and antipyretic drug (Cooper et al., 2017). Ingestion of APAP above the recommended dose can seriously damage liver tissue and even cause acute liver failure. According to the American acute liver failure research group, drug-induced liver injury in the US and most of Europe is largely caused by APAP overdose (Tujios and Lee, 2018). Many studies have concluded that the mechanism by which excessive APAP causes damage to hepatocytes is complicated but mainly involves oxidative stress, inflammatory cell infiltration, mitochondrial autophagy, and endoplasmic reticulum stress (Yang et al., 2017). Therefore, further exploration of the pathological mechanism associated with APAP-induced liver injury is urgently needed so that new therapeutic drugs can be developed.

Ginsenoside Rc is the main active component of ginseng and has become the main alternative for the treatment of liver diseases (Lee et al., 2019). We and others have previously confirmed the protective effects of Ginsenoside Rc against hepatitis and hepatic steatosis (Yang et al., 2020; Liang et al., 2021b). However, whether and how Ginsenoside Rc affects acute liver injury is far from clear. Using FXR knockout (FXR^−/−^) mice, mouse primary hepatocytes (MPHs), and RNA sequences, our current study is the first to show that the mechanism or pathway by which ginsenoside Rc reduces acetaminophen (APAP) overdose-induced hepatotoxicity involves the modulation of FXR-mediated antioxidative stress and anti-inflammatory and antiapoptotic functions.

Liver damage from APAP is likely to be caused by excessive n-acetylquinone imine (NAPQI) (Mohan et al., 2022) After a significant quantity of APAP has been ingested by the human body, it is processed through liver metabolism. APAP loses its activity by binding to sulfate and glucoside in the second stage of metabolism. A small portion of APAP is oxidized by cytochrome P450 enzymes (Glembotski et al., 2012). and converted to NAPQI, a highly active intermediate metabolite. Normally, NAPQI binds to glutathione (GSH) in the liver and is then degraded by the body. However, ingestion of a large amount of APAP bound with p-acetylphenol, sulfate, and glucosidic acid results in a significant quantity of APAP being oxidized by the P450 enzyme to produce a large quantity of NAPQI (Zhang Z. et al., 2022). Excessive NAPQI will consume a lot of glutathione in the liver. The remaining NAPQI reacts with the cell membrane, resulting in hepatocyte injury or death. Animal experiments have shown that liver glutathione consumption of greater than 70% will produce liver poisoning. With significant death of hepatocytes, oxidative stress and infectious inflammation in the liver are triggered (Cao et al., 2022). Oxidative stress and inflammation, in turn, aggravate liver damage, forming a vicious cycle (Elsayed et al., 2022). It can be seen that NAPQI is the key factor in liver injury caused by APAP. Inhibition of NAPQI synthase is also effective for treating APAP-induced liver injury. From our results, it can be found that Ginsenoside Rc can significantly reduce the synthesis of NAPQI synthase (Cyp2e, Cyp3a11, and Ugt1a11) in the liver injury model *in vivo* and *in vitro*, resulting in a decrease in the NAPQI content.

Mitochondrial protein, the key target of NAPQI, appears with excess APAP (Geib et al., 2021). Studies have shown that APAP can cause oxidative stress in the mitochondria and produce ROS through the percolation of electrons from the transport chain (Wang et al., 2021). Glutathione peroxidase (Glutathione Peroxidase, GSH-Px) and ATP synthase in mitochondria have been proven to bind to NAPQI (Kanno et al., 2017). When ATP synthase binds to NAPQI, GSH Px activity will decrease, resulting in the destruction of ATP synthesis and function. Mitochondrial complex I is a key target for ROS synthesis (Shi et al., 2021). In mice given APAP, the activity of mitochondrial complex I was found to increase accordingly (Barbier-Torres et al., 2017). There is an obvious positive relationship between the activity level of complex I and the degree of liver injury. Mitochondrial oxidative stress caused by APAP inevitably triggers cell death, which in turn produces an inflammatory response (Geib et al., 2021). Oxidative stress in the mitochondria during the metabolic phase increases their permeability while releasing mitochondrial proteins which causing apoptosis of the factor AIF (Shi et al., 2021). Mitochondrial proteins undergo nuclear translocation, which results in cell death and nuclear DNA fragmentation. In the current research, Ginsenoside Rc was shown to significantly reduce the level of NAPQI (Geib et al., 2021). Low levels of NAPQI promote the alleviation of liver cell apoptosis, oxidative stress, and the inflammatory response.

In the process of APAP-induced acute liver injury, superoxide free radicals are formed (Ullah et al., 2021). Once formed, they decompose into hydrogen peroxide (HO) and oxygen in mitochondria or are converted to peroxynitrite (ONOO-) through a reaction mechanism with endogenous nitric oxide (NO) (Ogino et al., 2021). Subsequently, HO can be cleared by GSH, catalase (CAT), glutathione peroxidase (GPX), peroxide reductase (PRX), and other antioxidant enzymes in hepatocytes (Li et al., 2022). At the same time, ONOO- formed in mitochondria reacts with GSH and is eliminated. These excess free radicals lead to GSH depletion, cause the accumulation of ONOO-, and form nitrotyrosine protein adducts, which eventually leads to mitochondrial DNA damage and hepatotoxicity (Chau et al., 2022). At this stage, N-acetylcysteine (NAC) a glutathione supplement, is the only antidote in clinical use for the treatment of APAP-induced DILI. It mainly enhances the detoxification effect of NAQPI by supplementing GSH. However, the drug still has some defects, such as a narrow treatment window (Lewis et al., 2022). Generally speaking, the adverse reactions of NAC are not life-threatening, but they include nausea, vomiting and allergic reactions. The detoxification effect of NAC is mainly effective 8 h before APAP poisoning, and then the treatment effect begins to decline obviously. Therefore, further research is needed on new drugs and potential targets that can treat APAP liver injury, especially for those patients who appear in the late stage of liver damage. Many studies on natural products extracted from foods have revealed a great deal of molecules that can be used to resist APAP hepatotoxicity (Subramanya et al., 2018). This study found that Ginsenoside Rc significantly increased the mRNA level of GSH synthase (Sod2, Gclm, Gclc), thus increasing the content of GSH. GSH can participate in a variety of key biochemical reactions, and its function is to protect the sulfhydryl groups of key enzymatic proteins from oxidation and loss of activity, so that they can be utilized by cells and maintain normal energy metabolism. At the same time, the combination of sulfhydryl and free radicals in the body can promote the reduction of free radicals, and then convert them into acidic substances, thereby increasing the excretion rate of free radicals and reducing the damage done to key organs by free radicals. The early signal of apoptosis is often a decrease in the GSH content, and subsequent oxygen free radicals will accelerate apoptosis.

Both chronic and acute liver disease are characterized by inflammation. However, the role of inflammation in liver disease is two-sided. On the one hand, inflammation can promote the removal of cell debris, which is conducive to the proliferation and repair of liver cells (Chen et al., 2018). On the other hand, when numerous hepatocytes die, inflammation is produced, resulting in liver injury (Fernandez-Checa et al., 2021). As the inflammatory reaction continues, cytokines are released to mediate the infiltration of inflammatory cells. The collected inflammatory cells can further kill hepatocytes over a short period of time, while long-term inflammation will induce the formation of liver fibrosis. (Chayanupatkul and Schiano, 2020). Ultimately, a variety of inflammatory factors participate in the injury of liver tissue, either directly or indirectly. As a key inflammatory factor, TNF-α not only accompanies the occurrence and development of DILI but also its content is also positively correlated with the severity of DILI (Navaei-Alipour et al., 2021). At the same time, TNF-α can induce hepatocyte apoptosis directly by mediating TNF-R1, or indirectly by inducing the production of a variety of cytokines, to form a complex inflammatory factor network that aggravates the progression of liver injury (Navaei-Alipour et al., 2021). In addition, interleukin-1 (IL-1) is also a strong pro-inflammatory factor. It can induce neutrophils and vascular endothelial cells to express adhesion molecules, trigger the secretion of other inflammatory factors, and participate in inflammatory responses (Navaei-Alipour et al., 2021). Secondly, IL-6 has a wide range of biological activities, which can promote the proliferation and differentiation of B cells, leading to the production of antibodies, increasing the formation of immune complexes as well as causing an inflammatory reaction (Navaei-Alipour et al., 2021). In conclusion, the excessive production of these inflammatory factors aggravates the damage done to liver pathological tissue. Many research results have described the activation of signaling molecules, such as NF-kB, MAPK and TXNIP/NLRP3, which participate in the inflammatory response and play important roles in liver injury. The levels of IL-1β, TNFα and NF-kB in hepatocytes are significantly and transiently increased following an overdose of APAP (Zhang C. et al., 2022). These elevated inflammatory factors promote the progression of the disease. In the current study, the levels of NAPQI, ROS, and inflammatory factors after APAP modeling differed from those of the normal group, which means the modeling was successful. After treatment with Ginsenoside Rc, the concentrations of ROS, and inflammatory factors (IL-1 β, TNF α, and NF-kB) decreased significantly, resulting in decreased liver apoptosis (Bax, Bcl2, and p53) and enhanced liver repair.

FXR not only balances bile acids, but also has significant regulatory effects on lipid metabolism, glucose metabolism and other related links in the liver (Molinaro and Marschall, 2022). It plays an important role in the development of chronic liver disease. Moreover, FXR forms a complex regulatory network through multiple signal pathways mediated by a cascade reaction signal pathway, G protein coupled receptor, cell surface receptor, and antigen receptor to protect the liver from damage due to pathogenic factors (Chiang et al., 2022). It was also confirmed that FXR can ensure that intestinal barrier function is maintained, preventing the formation of gallstones and reducing liver cholestasis, which may be based on the regulation of related genes affecting bile acid detoxification and drug metabolism by FXR (Norona et al., 2020). These genes mainly include phase I oxidases such as CYP3A4, phase II binding enzymes such as UGT2B4 and SULT2A1, and the phase I transporters MRP2 and ABCB4, which also affect the synthesis of NAPQI. From the above results, it can be seen that FXR may also affect the metabolic pathways of xenobiotics. At present, the FXR agonist 6-ECDCA, a therapeutic drug for primary biliary cirrhosis, has entered the clinical trial stage (Ho and Steinman, 2016). Similarly, another FXR agonist way-362450 is intended to treat nonalcoholic fatty liver and has also entered the clinical trial phase (Zhang et al., 2009). In a nutshell, FXR can enhance the synthesis of NAPQI, and reduce hepatic oxidative stress and the inflammatory response caused by DILI. Consistent with reports in the literature reports, our findings show that FXR can be used in the treatment of DILI. On the other hand, we also confirmed that Ginsenoside Rc, as an FXR agonist, plays a role in alleviating DILI. The detailed experimental evidence is as follows: 1) Molecular docking results showed that Ginsenoside Rc and FXR are well docked. 2) This study found that APAP leads to decreased expression of FXR and BSEP in the liver, but Ginsenoside Rc reverses this trend. BSEP can reduce the solubility of bile acids and eventually lead to cholestasis and liver injury. Some research suggests that BSEP is regulated by FXR. Activation of the FXR/BSEP signal axis can reduce oxidative stress and the inflammatory response in liver diseases such as cholestasis and liver cirrhosis. 3) The inhibition of APAP on SHP mRNA and protein expression can be reversed by ginsenoside Rc. The FXR-SHP axis can effectively regulate the metabolism of hepatic bile acids and lipids, liver immune inflammation, and tumor development. The efficacy of many FXR-SHP axis regulators has also been verified by clinical experiments. 4) We used FXR knockout mice and primary liver cells to clarify the therapeutic mechanism of ginsenoside Rc on APAP-induced liver injury using the APAP liver damage model *in vivo* and *in vitro*. The most compelling finding is that Ginsenoside Rc did not significantly reduce the APAP-metabolic enzymes, oxidative stress, and inflammatory response when FXR was knocked out *in vivo* or vitro.

Ginseng is used as a medicine and food worldwide. Many studies have confirmed that ginseng has pharmacological effects such as reducing the oxidative stress response, improving atherosclerosis, improving the osteoblast survival rate, and inhibiting tumor cell growth (Sarhene et al., 2021). Furthermore, many studies have shown that ginseng can promote glucose and lipid metabolism and improve liver function (Hou et al., 2022). Ginsenoside is the main effective substance contained in ginseng. Additionally, our research group has done a lot of work on ginsenoside’s effect on liver metabolism. Our previous works showed that Ginsenoside Rc can treat NAFLD by activating the SIRT6 protein (Yang et al., 2020); Ginsenoside Rb1 and Ginsenoside Rb3 can alleviate the liver glycolipid model. Those mechanisms are related to the reduction of oxidative stress and inflammatory response. The experiment also showed that ginsenoside Rc could improve the transcription level of SIRT1 (Supplementary Figure S5), and based on our research group, we added the KLF16 experiment. KLF16 is also a protein closely related to liver damage. The use of KLF16^−/−^ MPHs suggested that the transcription level of SIRT1 by ginsenoside Rc was not obvious, indicating that KLF16 may be the upstream target of ginsenoside Rc acting on SIRT1, which provides ideas for further study of ginsenoside Rc on liver damage in the future.

In conclusion, our study presents for the first time reported that Ginsenoside Rc could act as an effective FXR activator and treatment of acetaminophen (APAP) overdose-induced liver injury by modulating FXR to produce anti-oxidative stress, anti-inflammatory, and anti-apoptotic functions. However, in this study, the in-depth mechanism of Ginsenoside Rc treating liver damage through FXR has not been further discussed such as APAP adduct protein, mitochondrial autophagy and oxidative stress, and a detailed molecular mechanism experiment will be carried out in the future.

## Conclusion

In conclusion, our results provide direct evidence that Ginsenoside Rc can be considered a new FXR agonist that protects against APAP overdose-induced ALI in mice *via* the activation of FXR signaling. It works mainly by producing anti-inflammatory, antioxidant, and antiapoptotic effects. Our studies also confirmed that Ginsenoside Rc may be a promising compound for the treatment or relief of liver injury induced by APAP overdose.

## Data Availability

The original contributions presented in the study are included in the article/Supplementary Material, further inquiries can be directed to the corresponding authors.
