# Supplementary figures and images for: Ginsenoside Rc, as an FXR activator, alleviates acetaminophen-induced hepatotoxicity via relieving inflammation and oxidative stress
Source: Front Pharmacol. 2022 Oct 7;13:1027731. doi: 10.3389/fphar.2022.1027731 (PMC9585238; doi:10.3389/fphar.2022.1027731)

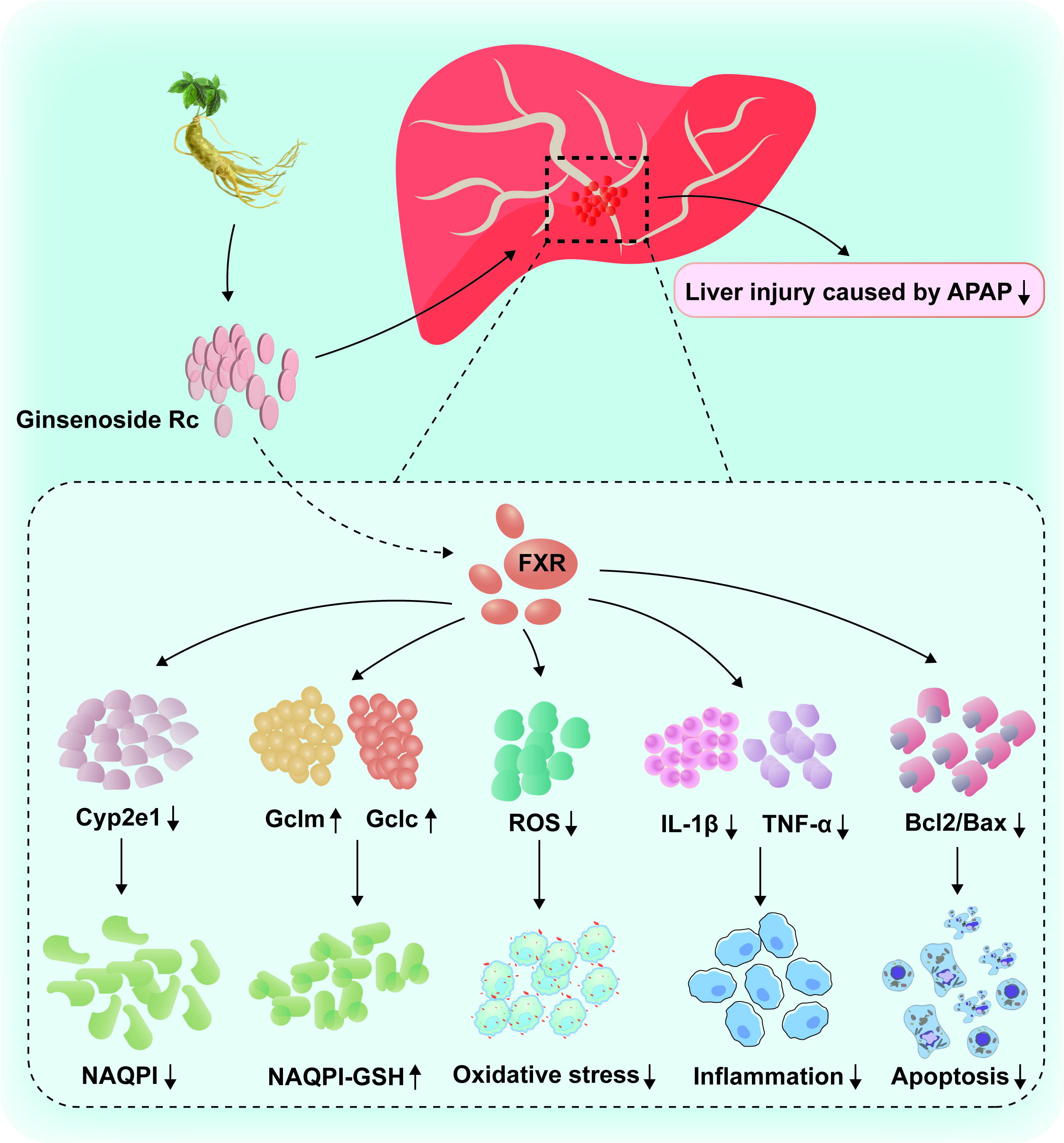

Supplement: Supplementary file 1 [file Image1.TIF]
