# Supplementary material for: Ginsenoside Rc, as an FXR activator, alleviates acetaminophen-induced hepatotoxicity via relieving inflammation and oxidative stress
Source: Front Pharmacol. 2022 Oct 7;13:1027731. doi: 10.3389/fphar.2022.1027731 (PMC9585238; doi:10.3389/fphar.2022.1027731)
Supplement: Supplementary file 2 [file DataSheet1.PDF]

Supplementary Material

1 Supplementary Table 1

Table 1

Primers used for Real-time PCR	Sequence (5' -> 3')	
Gene	Forward Primer	Reverse Primer
p53	CTCTCCCCCGCAAAAGAAAAA	CGGAACATCTCGAAGCGTTTA
Caspase	ATGGAGAACAACAAAACCTCAGT	TTGCTCCCATGTATGGTCTTTAC
Bax	TGAAGACAGGGGCCTTTTTG	AATTCGCCGGAGACACTCG
Bcl2	GTCGCTACCGTCGTGACTTC	CAGACATGCACCTACCCAGC
Sod2	CAGACCTGCCTTACGACTATGG	CTCGGTGGCGTTGAGATTGTT
Gclm	AGGAGCTTCGGGACTGTATCC	GGGACATGGTGCATTCCAAAA
Gclc	GGGGTGACGAGGTGGAGTA	GTTGGGGTTTGTCCTCTCCC
IL-1β	GCAACTGTTCCTGAACTCAACT	ATCTTTTGGGGTCCGTCAACT
IL-6	TAGTCCTTCCTACCCCAATTTCC	TTGGTCCTTAGCCACTCCTTC
Tnf-α	CCCTCACACTCAGATCATCTTCT	GCTACGACGTGGGCTACAG
Cyp2e1	CGTTGCCTTGCTTGTCTGGA	AAGAAAGGAATTGGGAAAGGTCC
Cyp3a11	CTGGAGGTGATGTTGAGTGTT	TGCTAGCCTAAGCATTGGAC
Ugt1a11	TCTGCTTCTTCCGTACCTTCT	GCTTCAGGTGCTATGACCACAA
Fxr	GCTTGATGTGCTACAAAAGCTG	CGTGGTGATGGTTGAATGTCC

Bsep	TCTGACTCAGTGATTCTTCGCA	CCCATAAACATCAGCCAGTTGT
Shp	TGGGTCCCAAGGAGTATGC	GCTCCAAGACTTCACACAGTG

1.1 Supplementary Figures

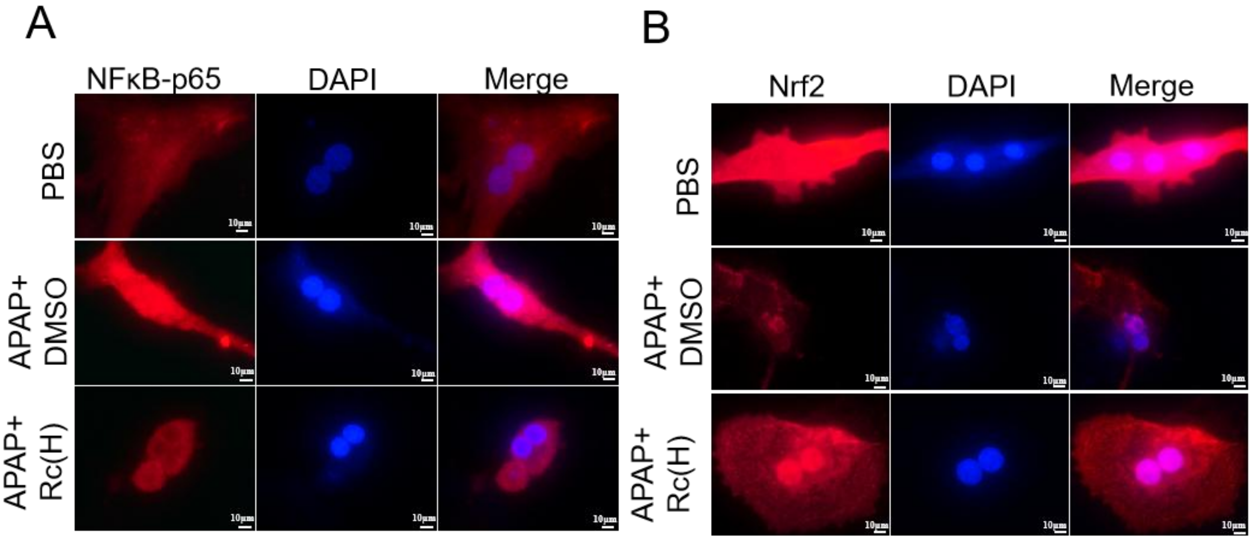

Supplementary Figure 1. Ginsenoside Rc administration significantly reduced NFκb levels and relieved oxidative stress in APAP-incubated MPHs **(A)** Immunofluorescence for NFkB (400×);
(A) Immunofluorescence for Nrf2 (400×);

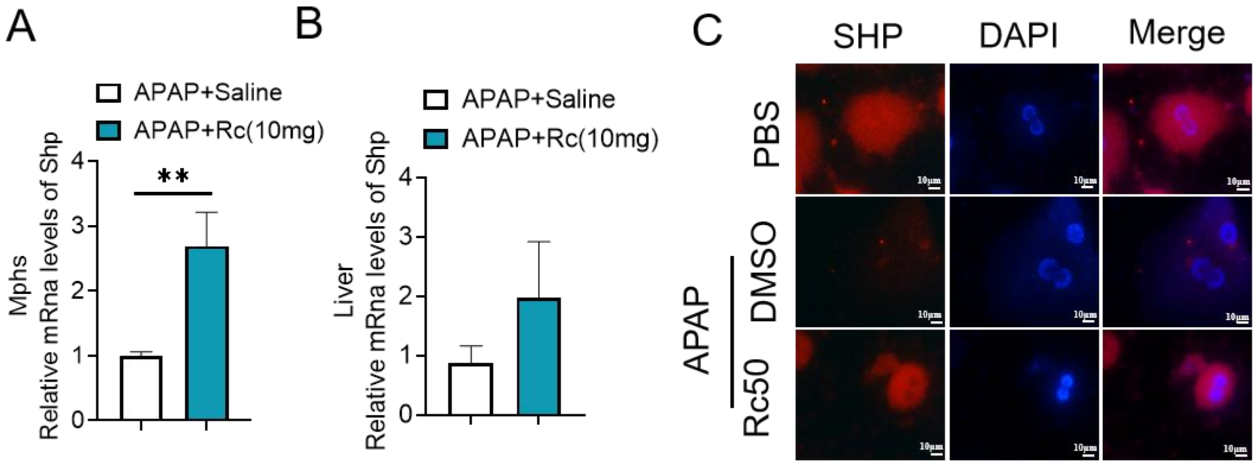

Supplementary Figures 2. Ginsenoside Rc increased the the expression of Shp (A) expression of mRNA levels of Shp in Mphs;(B) expression of mRNA levels of Shp in liver tissues;(C) Immunofluorescence for SHP (1000×); Data are means±SEM; n≥5/group. **P < 0.01

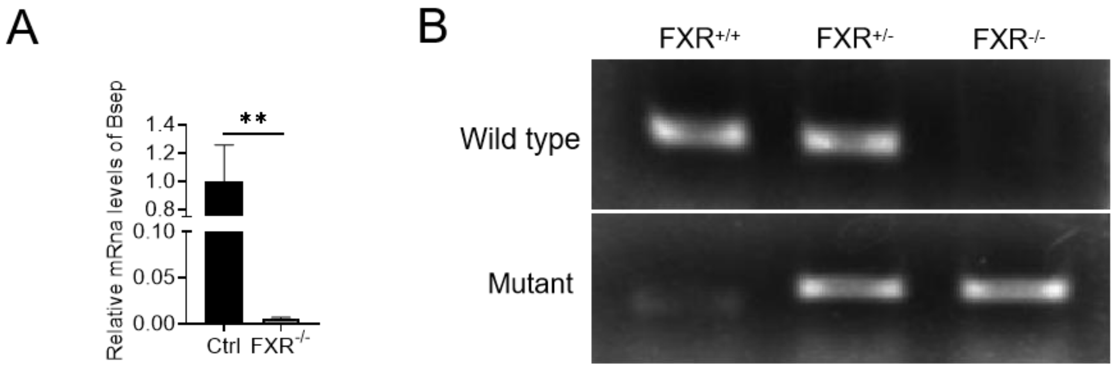

Supplementary Figures 3. Validation of FXR knockout mice. **(A)** Expression of mRNA levels of hepatic genes in Bsep; **(B)** Genotyping of FXR+/+ and FXR-/-; Data are means±SEM; n≥5/group. *P < 0.05,**P < 0.01

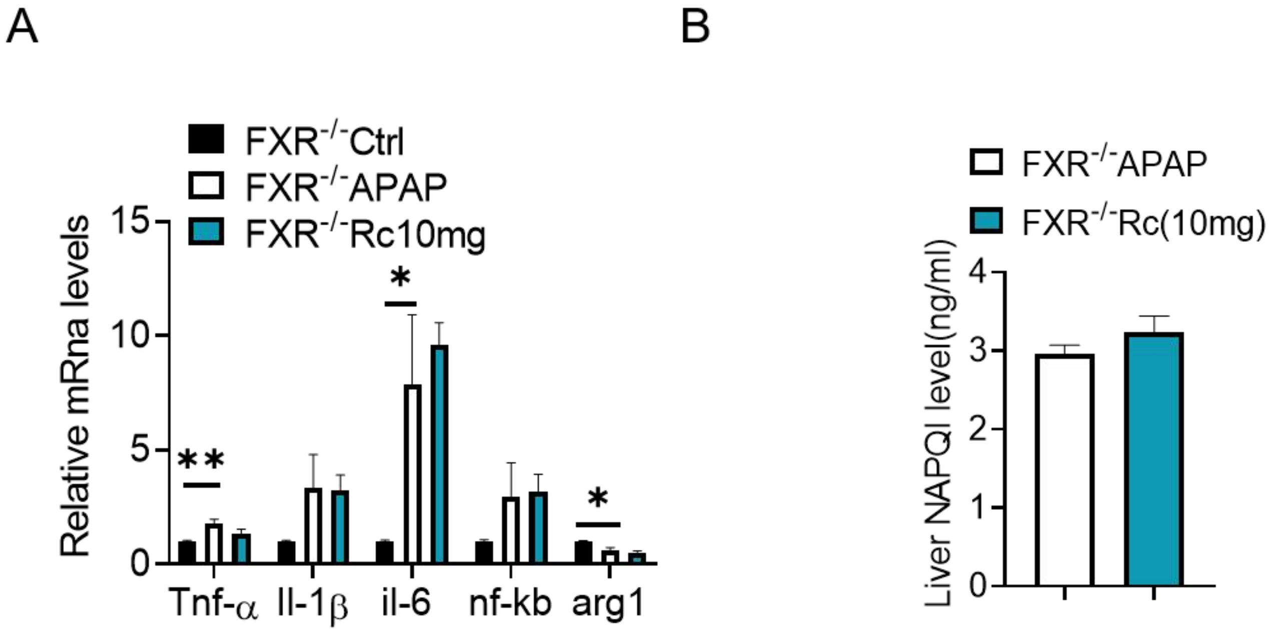

Supplementary Figures 4. Ginsenoside Rc alleviated inflammation and APAP metabolizing enzymes in ALi $FXR^{-/-}$mcie. **(A)** expression of mRNA levels of hepatic genes involved in inflmmation; **(B)** Liver levels of NAPQI; Data are means±SEM; n≥5/group. *P < 0.05,**P < 0.01.

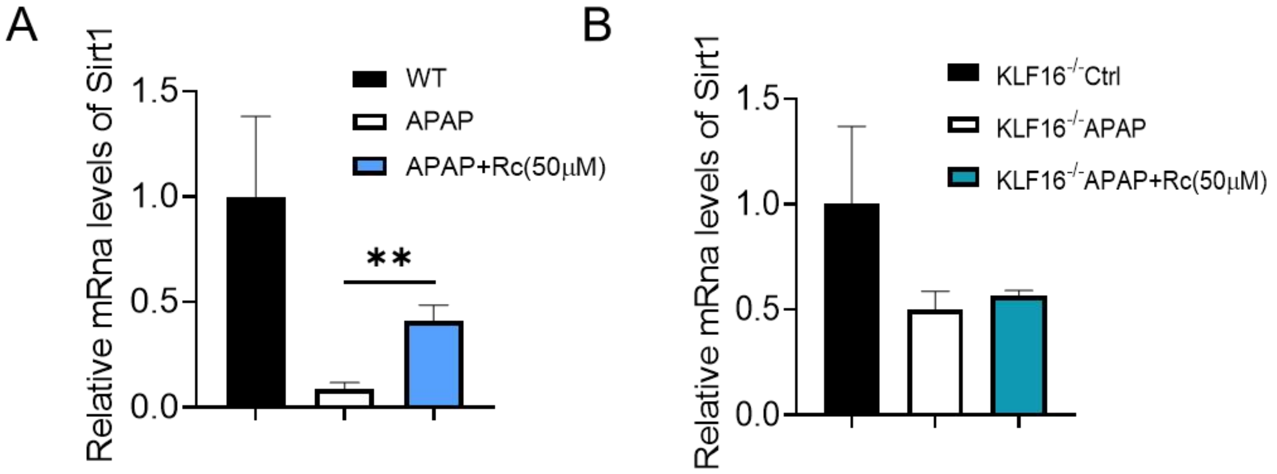

Supplementary Figures 5. ginsenoside Rc could improve the transcription level of SIRT1 **(A-B)** expression of mRNA levels of SIRT1; Data are means±SEM; n≥3/group.Data are means±SEM; n≥5/group. *P < 0.05,**P < 0.01.

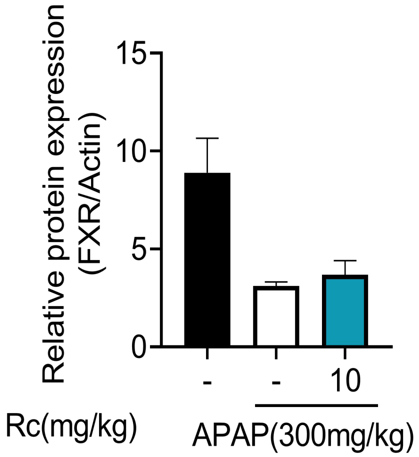

Supplementary Figures 6. The related gray value quantification for FIG. 4F.
